# Outcomes associated with remote monitoring without in-person evaluations for patients with cardiovascular implantable electronic devices

**DOI:** 10.1016/j.hroo.2025.07.021

**Published:** 2025-08-07

**Authors:** Laura T. Derry, Mary A. Whooley, Merritt H. Raitt, Thomas L. Rotering, Hui Shen, Gary Tarasovsky, Sanket S. Dhruva

**Affiliations:** 1 Department of Medicine, Stanford University, Stanford, California; 2 San Francisco Veterans Affairs Health Care System, San Francisco, California; 3 Section of General Internal Medicine, Department of Medicine, University of California, San Francisco School of Medicine, San Francisco, California; 4 Section of Cardiology, Department of Medicine, Portland Veterans Affairs Health Care System, Portland, Oregon; 5 Knight Cardiovascular Institute, Oregon Health and Sciences University, Portland, Oregon; 6 Section of Cardiology, Department of Medicine, University of California, San Francisco School of Medicine, San Francisco, California

**Keywords:** Cardiovascular implantable electronic devices, Implantable cardioverter-defibrillator, Pacemaker, Remote monitoring, Virtual care, Major adverse cardiac events, Veterans Health Administration

## Abstract

**Background:**

Traditionally, patients with cardiovascular implantable electronic devices (CIEDs) (pacemakers and implantable cardioverter-defibrillators) attend routine in-person evaluations at least annually, paired with remote monitoring (RM). Because similar data can be obtained through RM and in-person evaluations, it is unclear whether routine in-person evaluations are necessary.

**Objective:**

This study aimed to compare major adverse cardiac events (MACE) in patients who did vs did not receive in-person CIED care while participating in RM.

**Methods:**

We classified patients who received their CIED care within the Veterans Health Administration and sent ≥1 RM transmission between July 1, 2020, and June 30, 2021, into 2 groups based on clinician evaluation type for their CIED care: (1) at least 1 in-person evaluation or (2) remote-only evaluations. The primary outcome was MACE, a composite of all-cause mortality, stroke, and cardiac hospitalization, in the following year (July 1, 2021, to June 30, 2022). We performed multivariable logistic regression, adjusting for patient- and device-related characteristics.

**Results:**

Of 40,367 patients, 38,213 (94.7%) had at least 1 in-person evaluation for CIED care. The mean patient age was 72.8 years and 97.4% were male. There were 11,248 total MACEs (27.9%), 10,777 (28.2%) among patients who had at least 1 in-person evaluation and 471 (21.9%) among those with remote-only evaluations for CIED care. After multivariable adjustment, there was no significant difference in MACE (odds ratio 1.11; 95% confidence interval 0.98–1.25) between patients who had any in-person evaluation and those who had remote-only evaluations for CIED care.

**Conclusion:**

Among patients engaged in RM, the odds of MACE were similar regardless of whether patients had any in-person evaluations vs remote-only evaluations for CIED care.

**Key Findings**

▪ Among patients engaged in remote monitoring (RM), those receiving remote-only CIED care had fewer medical comorbidities and actionable findings on RM than those who also received in-person cardiovascular implantable electronic device (CIED) care.
▪ Major adverse cardiac events were similar among patients receiving remote-only vs in-person CIED care.
▪ These real-world findings support recent professional society consensus that routine in-person evaluations may not be necessary for patients consistently engaged in RM without recent alerts or high-risk clinical features.
▪ A remote-first CIED care model may improve device clinic efficiency and reduce potentially low-value visits for patients actively engaged in RM.

## Introduction

Cardiovascular implantable electronic devices (CIEDs), which include pacemakers and implantable cardioverter-defibrillators (ICDs), are permanently implanted in patients with or at risk of symptomatic and/or life-threatening cardiac arrhythmias.^1^, ^2^, ^3^, ^4^ After implantation, the standard of care for follow-up of patients with CIEDs has traditionally been routine in-person evaluations, which usually occur at least annually, paired with remote monitoring (RM). RM is the transmission of CIED data using cellular, Wi-Fi, or analog transmission from a patient’s residence.^5^ RM has a class 1, level of evidence A Heart Rhythm Society recommendation^6^ because it enables earlier detection and clinical action for cardiac arrhythmias or CIED malfunction, thereby improving cardiovascular outcomes, including reductions in mortality, hospitalizations, and ICD shocks.^7^^,^^8^ RM also improves patient satisfaction, in large part because it can enable fewer in-person clinic visits.^9^^,^^10^

Although RM is central to CIED care, it has historically been viewed as a complement—but not a substitute—for routine in-person evaluations, even though the information obtained through RM is nearly identical to that obtained through in-person evaluations. However, the 2023 Heart Rhythm Society Expert Consensus Statement on practical management of the remote device clinic indicated that the future of CIED care would include fewer routine in-person evaluations. The consensus states that it is reasonable to reduce the frequency of routine in-person evaluations for patients with consistent and continuous RM connectivity who have neither had recent alerts nor other cardiac comorbidities, with visits scheduled when device alerts prompt a need for in-person evaluation.^6^

Two randomized controlled trials (RCTs) conducted outside the United States have found no difference in clinical outcomes without routine in-person evaluations for patients engaged in RM.^11^^,^^12^ However, these RCTs were conducted in small international patient populations. Therefore, real-world evaluation would be informative to determine whether remote-only care is associated with similar outcomes compared with routine in-person evaluations for CIED care plus RM.

One study of 1937 patients with CIEDs actively engaged in RM at a single center in Italy whose routine in-person evaluations were changed to an RM-only model over a 3-year period found time savings for clinicians, a reduction in follow-up visits, and no increase in adverse outcomes.^13^ However, there is a need for larger studies to evaluate cardiovascular outcomes associated with a remote-only approach before a larger shift in care can occur. Accordingly, we sought to determine whether patients with remote-only evaluations had similar outcomes to patients who also had any in-person evaluation for their CIED care among patients receiving care in the United States’ largest public integrated health system, the Veterans Health Administration (VHA).

## Methods

This quality improvement project was conducted in accordance with the Department of Veterans Affairs Office of Research & Development Program Guide 1200.21 “VHA (Veterans Health Administration) Operations Activities That May Constitute Research”; patient consent was not obtained because this was part a quality improvement study to assess and improve the quality of care for veterans with CIEDs and did not require institutional review board approval.

### Data sources

All patients who receive CIED care within VHA and participate in RM are scheduled to send remote transmissions every 90 days, along with patient-initiated and alert-initiated transmissions.^14^ All of these RM transmissions are reviewed centrally by clinicians at the Veterans Affairs National Cardiac Device Surveillance Program (VANCDSP) within 1 business day for alert transmissions and 3 business days for nonalert transmissions. If any potentially clinically actionable findings are identified, the responsible VHA device clinic (1 of 122) is notified immediately and takes clinical action as needed (there is no direct feedback to patients except through contact from their local clinicians). The VANCDSP was our first data source. Our second data source was the VHA Corporate Data Warehouse (CDW), a national database that contains patient-level electronic health record data, including demographics, medical history, and inpatient, emergency department, and outpatient (including telephone and video) evaluation records. The VHA CDW also includes claims paid by VHA for care provided by non-VHA clinicians. Our third data source was Centers for Medicare and Medicaid Services (CMS) data, which includes information on inpatient and outpatient evaluations outside of the VHA for veteran patients with CMS coverage. We linked VANCDSP, CDW, and CMS data for this study.

### Patient population

**Figure 1:**
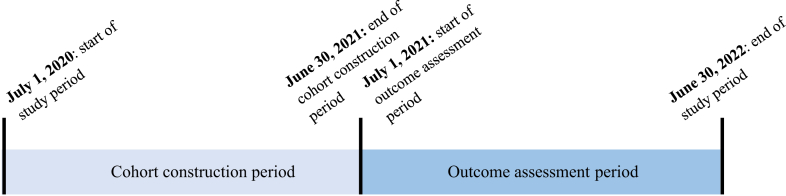
Diagram of study period (July 1, 2020, and June 30, 2022), which included a cohort construction period (July 1, 2020, to June 30, 2021) followed by a separate outcome assessment period (July 1, 2021, to June 30, 2022).

We identified veterans with pacemakers or ICDs who received their CIED care within the VHA system and had agreed to RM between July 1, 2020, and June 30, 2022, which was divided into a cohort construction period (July 1, 2020, to June 30, 2021) and a separate outcome assessment period (July 1, 2021, to June 30, 2022) (Figure 1). Patients were included if their CIED was implanted before the start of the cohort construction period, they were actively monitored by the VANCDSP with no status changes (which means that they were alive during this cohort construction period, did not receive care provided by non-VHA clinicians for their CIED, and were actively enrolled in RM during this period), and they sent at least 1 RM transmission during this cohort construction period (Supplemental Tables 1 and 2). Patients were also excluded if they received hospice care (Supplemental Table 3), died during the cohort construction period, were followed at 1 of 2 VHA facilities that transitioned to a new electronic health record during the study period, had missing RM adherence data, or could not be linked between the VANCDSP and CDW data sources.

### Classification based on evaluations for CIED care

Patients—all of whom sent at least 1 RM transmission during the cohort construction period—were classified into 1 of 2 study groups based on the evaluation type for CIED care during the cohort construction period. The first group included patients who received any in-person evaluation for CIED care. Receipt of in-person care was determined based on the presence of Current Procedural Terminology (CPT) codes (93260, 93261, 93279, 93280, 93281, 93282, 93283, 93284, 93286, 98287, 93288, or 93289) or VHA stop codes indicating in-person evaluations, clinical video telehealth (CVT) (in which a clinician at 1 VHA facility provides care via video to a patient at another VHA facility), and/or inpatient hospitalizations in addition to RM. The second group included patients who did not have any of these evaluations (in-person, CVT, inpatient hospitalization) for CIED care and only sent RM transmissions. Patients in this group could have had virtual visits for CIED care, but they did not have any in-person visits. In-person and CVT evaluations were defined based on a CPT code for in-person evaluation (Supplemental Table 2).

### Outcomes

The primary outcome was major adverse cardiac events (MACEs), defined as a composite of all-cause mortality, stroke, and cardiac hospitalization. These outcomes were selected because theoretically identifying and managing active cardiac conditions at in-person evaluations for CIED care could reduce the incidence of these events. Cardiac hospitalization was identified based on International Classification of Diseases, Tenth Revision, diagnosis codes for primary discharge diagnoses of heart failure, cardiac arrhythmias, cardiac arrest, syncope/collapse, and ischemic heart disease using VHA CDW (including both within and outside VHA), Medicare (Part A, Medicare Provider Analysis and Review file), Medicare Part C (Encounter files), and Medicaid data (Supplemental Table 4).

### Covariates

Multiple patient- and device-related characteristics were used for risk adjustment. Patient characteristics were demographics, the Charlson comorbidity index,^15^ and inpatient and outpatient health care utilization. Demographic characteristics were assessed on July 1, 2020, except for distance to primary care, which was assessed for the year 2019 since the start of the cohort construction period was midyear (July 1, 2020). Comorbidities were defined as at least 2 outpatient or at least 1 inpatient diagnosis code between July 1, 2018, and June 30, 2021 (Supplemental Tables 5 and 6). Inpatient clinical care utilization was measured by identifying cardiac hospitalizations by primary discharge diagnosis (Supplemental Table 4). Long-term care utilization and outpatient cardiology clinic evaluations were identified by Healthcare Common Procedure Coding System codes and VHA stop codes (which identify clinical services provided) (Supplemental Table 7). Device characteristics were CIED type (pacemaker or ICD), presence of cardiac resynchronization therapy, manufacturer, wireless-RM capability, device on alert and/or recall, number of RM transmissions, and RM adherence. RM adherence was calculated by determining whether the patient’s last transmission occurred within the prescribed transmission interval (usually 90 days for all patients) plus a 10-day buffer period in case of late transmissions for reasons such as travel.^16^ If an RM transmission had been sent within that interval, the patient was considered adherent. We determined the adherence percentage as the number of days adherent divided by the total days of follow-up, using a standardized 400-day interval used by the VANCDSP. The interval used for the follow-up period was July 1, 2020 to August 5, 2021, to align with the study period. Finally, we adjusted for abnormalities identified on RM transmissions that could lead to clinical action, including arrhythmias (eg, atrial fibrillation), possible generator or lead-related malfunction, and ICD therapy (either antitachycardia pacing or shock) for patients with ICDs (Supplemental Table 8).

### Statistical analysis

Descriptive statistics, stratified by the 2 patient groups, are presented for patient- and device-related characteristics. Differences were assessed using χ^2^ tests for categorical variables and *t* tests for continuous variables. Next, using a multivariable logistic regression model, we examined the primary outcome and its components (mortality, stroke, and cardiac hospitalization) by adjusting for demographics, device factors (device abnormality, clinically significant arrhythmias, and ICD therapy [including antitachycardia pacing and shocks] detected on RM, RM adherence, and number of RM transmissions), geographic factors (rural residence, distance to tertiary care, and region), comorbidities, long-term care utilization, and inpatient health care utilization during the cohort construction period. Statistical significance was based on a 2-sided *P* < .05. Statistical analyses were performed using SAS Enterprise Guide 8.2 (SAS Institute Inc).

## Results

**Figure 2:**
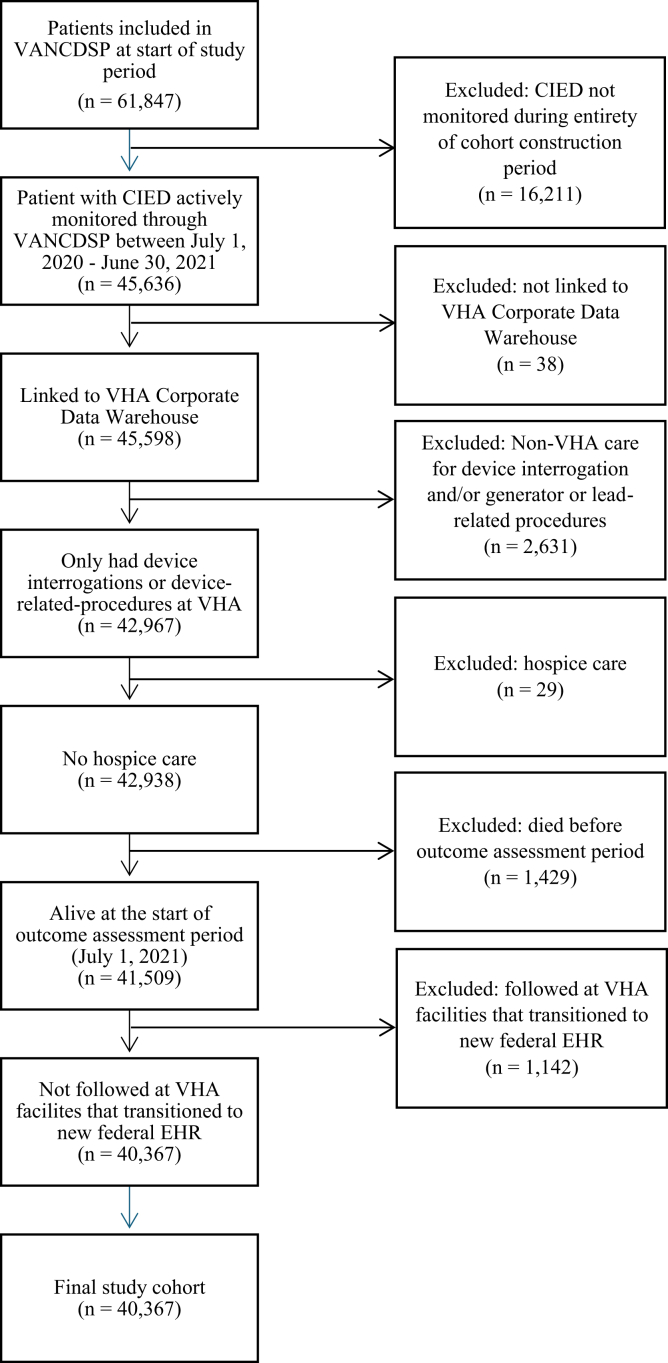
Cohort construction of veterans with a CIED who were actively enrolled and remotely monitored in the VANCDSP between July 1, 2020, and June 30, 2021. CIED = cardiac implantable electronic device; EHR = electronic health record; VANCDSP = Veterans Affairs National Cardiac Device Surveillance Program; VHA = Veterans Health Administration.

The cohort included 40,367 patients with CIEDs participating in RM (Figure 2), including 38,213 (94.7%) who had at least 1 in-person evaluation for CIED care and 2154 (5.3%) who did not have an in-person evaluation during the cohort construction period (Table 1).

**Table 1:** Baseline patient characteristics

Variable	Total (N = 40,367)	Any in-person evaluation (n = 38,213)	Remote-only evaluations (n = 2154)	*P* value
Age on July 1, 2020 (start of the cohort construction period), y, mean ± SD	72.8 ± 10.0	72.9 ± 9.9	72.0 ± 10.8	<.001
Male sex, mean ± SD	39,327 (97.4%)	37,228 (97.4%)	2099 (97.4%)	.97
Hispanic or Latino ethnicity∗, mean ± SD	1464 (3.6%)	1344 (3.5%)	120 (5.6%)	<.001
Race∗, n (%)				.01
American Indian or Alaska Native	300 (0.7)	277 (0.7)	23 (1.1)	
Asian	180 (0.4)	163 (0.4)	17 (0.8)	
Black or African American	5884 (14.6)	5600 (14.7)	284 (13.2)	
Native Hawaiian or other Pacific Islander	297 (0.7)	281 (0.7)	16 (0.7)	
White	31,228 (77.4)	29,615 (77.5)	1613 (74.9)	
Marital status∗, n (%)				<.001
Married	22,746 (56.4)	21,577 (56.5)	1169 (54.3)	
Divorced/separated	11,017 (27.3)	10,380 (27.2)	637 (29.6)	
Never married/single	2913 (7.2)	2739 (7.2)	174 (8.1)	
Window/widower	3581 (8.9)	3419 (8.9)	162 (7.5)	
Rural (vs urban) residence, n (%)	15,088 (37.4)	14,311 (37.5)	777 (36.1)	.21
Region of VHA facility providing patient’s CIED care∗, n (%)				<.001
East North Central	5090 (12.6)	4926 (12.9)	164 (7.6)	
East South Central	3238 (8.0)	3089 (8.1)	149 (6.9)	
Mid-Atlantic	2287 (5.7)	2241 (5.9)	46 (2.1)	
Mountain	4023 (10.0)	3841 (10.1)	182 (8.4)	
New England	1523 (3.8)	1503 (3.9)	20 (0.9)	
Pacific	3967 (9.8)	3274 (8.6)	693 (32.2)	
South Atlantic	10,181 (25.2)	9995 (26.2)	186 (8.6)	
West North Central	3892 (9.6)	3739 (9.8)	153 (7.1)	
West South Central	6127 (15.2)	5568 (14.6)	559 (26.0)	
Distance to nearest primary care∗, n (%)				.05
≤25 miles	32,088 (79.5)	30,345 (79.4)	1743 (80.9)	
26–50 miles	6824 (16.9)	6504 (17.0)	320 (14.9)	
51–100 miles	1211 (3.0)	1138 (3.0)	73 (3.4)	
>100 miles	118 (0.3)	110 (0.3)	8 (0.4)	
Distance to nearest specialty care∗, n (%)				<.001
≤25 miles	18,113 (44.9)	17,186 (45.0)	927 (43.0)	
6–50 miles	10,443 (25.9)	9933 (26.0)	510 (23.7)	
51–100 miles	8714 (21.6)	8217 (21.5)	497 (23.1)	
>100 miles	2971 (7.4)	2760 (7.2)	211 (9.8)	
Distance to nearest tertiary care∗^,^†, n (%)				<.001
≤25 miles	10,255 (25.4)	9606 (25.1)	649 (30.1)	
26–50 miles	7561 (18.7)	7152 (18.7)	409 (19.0)	
51–100 miles	10,198 (25.3)	9705 (25.4)	493 (22.9)	
>100 miles	12,222 (30.3)	11,628 (30.4)	594 (27.6)	
Comorbidities				
Cardiovascular comorbidities, n (%)				
Atrial fibrillation	20,721 (51.3)	19,861 (52.0)	860 (39.9)	<.001
Coronary artery bypass graft	578 (1.4)	560 (1.5)	18 (0.8)	.02
Dyslipidemia	33,002 (81.8)	31,419 (82.2)	1583 (73.5)	<.001
Heart failure	22,727 (56.3)	21,689 (56.8)	1038 (48.2)	<.001
Heart transplant recipient	56 (0.1)	53 (0.1)	3 (0.1)	.99
Hypertension	36,156 (89.6)	34,364 (89.9)	1792 (83.2)	<.001
Ischemic heart disease	26,756 (66.3)	25,492 (66.7)	1264 (58.7)	<.001
Left ventricular assist device	58 (0.1)	56 (0.1)	2 (0.1)	.52
Percutaneous coronary intervention	1970 (4.9)	1877 (4.9)	93 (4.3)	.21
Previous myocardial infarction	8701 (21.6)	8351 (21.9)	350 (16.2)	<.001
Previous ventricular tachycardia	7970 (19.7)	7688 (20.1)	282 (13.1)	<.001
Valvular heart disease	8288 (20.5)	8005 (21.0)	283 (13.1)	<.001
Other comorbidities, n (%)				
Alcohol use disorder	2821 (7.0)	2698 (7.1)	123 (5.7)	.02
Cancer (excluding nonmelanoma skin cancers)	6702 (16.6)	6422 (16.8)	280 (13.0)	<.001
Chronic kidney disease	18,411 (45.6)	17,693 (46.3)	718 (33.3)	<.001
Chronic obstructive pulmonary disease	11,619 (28.8)	11,104 (29.1)	515 (23.9)	<.001
Diabetes mellitus	20,018 (49.6)	19,059 (49.9)	959 (44.5)	<.001
End-stage renal disease	923 (2.3)	889 (2.3)	34 (1.6)	.02
Interstitial lung disease	907 (2.2)	877 (2.3)	30 (1.4)	.06
Peripheral vascular disease	7034 (17.4)	6729 (17.6)	305 (14.2)	<.001
Stroke or transient ischemic attack	4312 (10.7)	4126 (10.8)	186 (8.6)	.002
CCI				
CCI score, mean ± SD	4.1 ± 2.9	4.1 ± 2.9	3.3 ± 2.6	<.001
CCI severity, n (%)				
CCI score 0	3559 (8.8)	3270 (8.6)	289 (13.4)	<.001
Mild (CCI score 1–2)	10,470 (25.9)	9766 (25.6)	704 (32.7)	
Moderate (CCI score 3–4)	10,073 (25.0)	9545 (25.0)	528 (24.5)	
Severe (CCI score ≥5)	16,261 (40.3)	15,628 (40.9)	633 (29.4)	
Health care utilization between July 1, 2018, and June 30, 2020, n (%)				
Long-term care utilization	1459 (3.6)	1380 (3.6)	79 (3.7)	.89
Cardiac hospitalization	10,211 (25.3)	9786 (25.6)	425 (19.7)	<.001
Outpatient cardiac care	39,310 (97.4)	37,223 (97.4)	2087 (96.9)	.14

CCI = Charlson comorbidity index; CIED = cardiac implantable electronic device; SD = standard deviation; VHA = Veterans Health Administration.

∗ Totals may not equal 100% because of the following numbers of unknown/missing variables: race (n = 2474), ethnicity (n = 1679), marital status (n = 106), region (n = 39), distance to nearest primary care (n = 126), distance to nearest specialty care (n = 126), and distance to nearest tertiary care (n = 131).

† States by region: New England (CT, ME, MA, NH, RI, VT), Mid-Atlantic (NJ, NY, PA), South Atlantic (DE, DC, FL, GA, MD, NC, SC, VA, WV), East North Central (IN, IL, MI, OH, WI), East South Central (AL, KY, MS, TN), West North Central (IA, NE, KS, ND, MN, SD, MO), West South Central (AR, LA, OK, TX), Mountain (AZ, CO, ID, NM, MT, UT, NV, WY), and Pacific (AK, CA, HI, OR, WA).

### Patient demographics and clinical characteristics

Overall, the mean patient age was 72.8 years (standard deviation [SD] 10.0), and 97.4% were male (Table 1). The percentage of female patients was the same across both groups, but patients in the remote-only group were approximately 1 year younger than those in the group with 1 or more in-person evaluations (mean [±SD] 72.0 ± 10.8 vs 72.9 ± 9.9, respectively; *P* < .001). The percentage of rural patients was similar between both groups (37.5% and 36.1% for in-person and remote-only, respectively; *P* = .21). Patients in the remote-only group were less likely to have comorbidities, including significantly lower rates of atrial fibrillation, heart failure, ischemic heart disease, cancer, peripheral vascular disease, and valvular heart disease (all with *P* < .001). Patients in the remote-only group were also less likely to be hospitalized for cardiac reasons in the 2 years before the cohort construction period (19.7% and 25.6% for remote-only and in-person, respectively; *P* < .001). Although patients in the group with remote-only evaluations for CIED care did not have an in-person evaluation for their CIED during the cohort construction period, 96.9% had received outpatient cardiac care (including general cardiology) during the previous 2 years.

### Device-related characteristics

There was a slightly higher percentage of patients with pacemakers (51.4%) than ICDs (48.6%) in the study population (Table 2), but this difference was not statistically significant between the 2 groups. A lower percentage of patients with cardiac resynchronization therapy devices was seen in the remote-only group compared with those who had in-person evaluations for CIED care (16.6% vs 21.3%, respectively; *P* < .01). The mean (±SD) number of RM transmissions with a detected arrhythmia was lower among patients with no in-person evaluation for CIED care (3.0 ± 3.8 vs 3.8 ± 5.7; *P* < .001), generator or lead abnormalities (0.5 ± 1.5 vs 0.7 ± 2.4; *P* < .001), and ICD therapy (0.15 ± 1.0 vs 0.23 ± 1.4; *P* < .001). The mean (±SD) RM adherence was not significantly different between the 2 groups (86.2% ± 21.9% vs 88.5% ± 19.7% for remote-only and in-person, respectively; *P* = .06).

**Table 2:** Device-related characteristics of CIEDs among veterans participating in RM

Variable	Total (N = 40,367)	In-person (n = 38,213)	Remote-only (n = 2154)	*P* value
Device type, n (%)				.79
ICD	19,604 (48.6)	18,564 (48.6)	1040 (48.3)	
Pacemaker	20,763 (51.4)	19,649 (51.4)	1114 (51.7)	
CRT, n (%)	8486 (21.0)	8128 (21.3)	358 (16.6)	<.001
Manufacturer, n (%)				<.001
Abbott	8633 (21.4)	8257 (21.6)	376 (17.5)	
Biotronik	2519 (6.2)	2387 (6.2)	132 (6.1)	
Boston Scientific (includes Guidant)	8849 (21.9)	8849 (21.9)	8849 (21.9)	
LivaNova (includes ELA Medical and Sorin Group)	8 (0.0)	8 (0.0)	0 (0.0)	
Medtronic	20,358 (50.4)	19,173 (50.2)	1185 (55.0)	
Wireless capable, n (%)	32,447 (80.4)	30,791 (80.6)	1656 (76.9)	<.001
Device on alert and/or recall,∗ n (%)	22,624 (56.0)	21,437 (56.1)	1187 (55.1)	.37
Transmissions with clinically significant arrhythmia detected on RM,∗ mean ± SD	3.7 ± 5.6	3.8 ± 5.7	3.0 ± 3.8	<.001
Transmissions with device abnormality detected on RM,∗ mean ± SD	0.67 ± 2.3	0.67 ± 2.4	0.46 ± 1.5	.002
Transmissions with ICD therapy detected on RM,∗ mean ± SD	0.23 ± 1.4	0.23 ± 1.4	0.15 ± 0.97	<.001
RM adherence,∗ mean % ± SD	88.4 ± 19.8	88.5 ± 19.7	86.2 ± 21.9	.06
Number of RM Transmissions,∗ mean ± SD	6.2 ± 5.7	6.2 ± 5.7	5.6 ± 5.2	<.001

CRT = cardiac resynchronization therapy; ICD = implantable cardioverter-defibrillator; RM = remote monitoring; SD = standard deviation.

∗ Assessed during the cohort construction period (July 1, 2020, to June 30, 2021).

### Clinical outcomes

There were 11,248 total MACEs (27.9%), 10,777 (28.2%) among patients who had at least 1 in-person evaluation and 471 (21.9%) among those with remote-only evaluations for CIED care (Table 3). After multivariable adjustment in the full model, patients who had at least 1 in-person evaluation did not have a significantly different odds of MACE (odds ratio [OR] 1.11; 95% confidence interval [CI] 0.98–1.25) than those with remote-only evaluations for CIED care (Table 4). Furthermore, there was no statistically significant association between having at least 1 in-person evaluation and the component outcomes of all-cause mortality (OR 1.04; 95% CI 0.88–1.23) or stroke (OR 0.75; 95% CI 0.52–1.08). However, the group with at least 1 in-person evaluation was associated with higher odds of cardiac hospitalization (OR 1.20; 95% CI 1.04–1.38) than remote-only CIED evaluations.

**Table 3:** Primary outcome events during outcome assessment period, July 1, 2021 to June 30, 2022

Primary outcome events, n (%)	Total (N = 40,367)	In-person (n = 38,213)	Remote-only (n = 2154)	*P* value∗
All-cause mortality	4043 (10.0)	3863 (10.1)	180 (8.4)	.008
Cardiac hospitalization	6635 (16.4)	6378 (16.7)	257 (11.9)	<.001
Stroke	570 (1.4)	536 (1.4)	34 (1.6)	.50
Total major adverse cardiac events	11,248 (27.9)	10,777 (28.2)	471 (21.9)	

∗ *P* values reflect unadjusted comparisons between groups.

**Table 4:** Primary outcome analysis during outcome assessment period, July 1, 2021 to June 30, 2022

Primary outcome analysis, odds ratio (95% confidence interval)	Unadjusted analysis	Fully adjusted model∗
All-cause mortality	1.23 (1.05–1.44)	1.04 (0.88–1.23)
Cardiac hospitalization	1.48 (1.29–1.69)	1.20 (1.04–1.38)
Stroke	0.89 (0.63–1.26)	0.75 (0.52–1.08)
Composite MACE	1.35 (1.20–1.50)	1.11 (0.98–1.25)

MACE = major adverse cardiac event.

∗ Adjusted for demographic characteristics, geographic region, rural residence, distance to tertiary care, Charlson comorbidity index, long-term care utilization, cardiac hospitalization (July 2018 to June 2020), device abnormality, clinically significant arrhythmias, and implantable cardioverter-defibrillator therapy (including antitachycardia pacing and shocks) detected on remote monitoring (RM), RM adherence, and number of RM transmissions.

## Discussion

Among more than 40,000 patients engaged in RM in the United States’ largest public integrated health care system, the odds of MACE were not significantly different regardless of whether patients had any in-person vs remote-only evaluations for CIED care. The similarity in outcomes may reflect the dominant effect of RM; in-person evaluations may not offer additional benefit when RM has high, reliable adherence. Our real-world study had a large number of events, and the narrow CIs provide confidence in our findings. Although only 5.3% of patients had no in-person evaluations for CIED care, these results represent more than 2000 patients and build on multiple published RCTs^11^^,^^12^ and smaller observational studies^13^^,^^17^ to suggest that routine in-person evaluations for CIED care may not improve outcomes and, thus, may not be necessary for patients who are actively participating in RM. Patients, clinicians, and health care systems could likely benefit by reducing these routine evaluations and refocusing resources on CIED care evaluations that are more likely to yield actionable interventions because they are prompted by abnormalities on RM transmissions.^18^

Although RM has been the standard of care for CIED follow-up since its development and provides detailed clinical information, patients have still traditionally attended at least an annual routine in-person follow-up.^5^ Although 2 RCTs demonstrated similar clinical outcomes between routine in-person evaluations and RM and were complemented by single-center experience in Italy and Finland,^11^^,^^12^^,^^17^^,^^19^ these may have limited generalizability to other settings. Based on these and previous RCTs, cardiac electrophysiology professional societies now state that in-person follow-up is reasonable every 24 months for patients actively engaged in RM (with consistent and continuous RM connectivity who have neither had recent alerts nor other cardiac comorbidities), which is a significant lengthening of the previous interval. Our observational findings support this recommendation, demonstrating statistically similar odds of MACE among patients actively engaged in RM who had no in-person evaluations for CIED care over a 1-year period. Furthermore, the professional society consensus suggests that routine scheduled evaluations may be “low-value visits,” and device clinics could optimize workflows to focus on actionable events and high-yield interventions.^6^ Our observational findings support this idea. A recent study found that cumulative staff time for in-person evaluations ranged from 37.8 to 51 minutes compared with a much shorter mean cumulative staff time required to review an RM transmission ranging from 9.4 to 13.5 minutes (16.1–21.7 minutes for actionable events).^20^ This highlights efficiency and workflow opportunities for device clinics that could allow them to refocus staff time and effort on other clinical indications and conditions.

Although barriers exist and will need to be addressed to implement a remote-first care strategy, the remote-first care model has the potential to reduce low-value evaluations, improve access to device clinics and timeliness of care, and improve patient and clinician satisfaction by focusing on care that is more likely to be needed. Furthermore, targeted strategies to improve patient adherence to RM^21^ and new technologies that allow continuous connectivity^22^ can facilitate a remote-first care strategy. Adherence to RM and continuous connectivity are essential to ensure that clinically actionable events are identified and intervened upon in a timely manner in a remote-first care model.^23^ To implement such a remote-first strategy, health systems and device clinics will need to ensure that the appropriate resources are in place to support these models and that patients and clinicians are educated about it.^18^ Furthermore, patients should routinely follow up, as appropriate, with their primary and other specialty care clinicians (eg, general cardiologists) to ensure they are up to date for non-CIED-related care.

Future prospective studies after implementation should evaluate patient and clinician perspectives on remote-first care models, such as impacts on device clinic workflows and quality of and satisfaction with care. A recent study assessed patient and clinician perspectives on alert-based RM-first care strategies,^18^ but these outcomes should be evaluated prospectively in future research along with cost-effectiveness. Adequate staffing, data and alert management, and reimbursement reform are all considerations that must be tailored at the device clinic and system level to make this care model successful. Recent studies have demonstrated that the increasing volume of data, including nonactionable data, from both in-person and remote interrogations has a significant impact on CIED monitoring programs.^17^^,^^24^^,^^25^ Indeed, the Patient Centered Outcomes Research Institute recently funded a $30 million trial of alert-based care for CIEDs, which will provide both clinical outcomes data and clinician and patient perspectives.^26^

Our findings must be considered in the context of their limitations. First, this study was conducted in a single, integrated health care system, the VHA, and the findings may not be generalizable to other health systems. The VHA is unique because it is both a health care provider and payer and has RM care standardized through its centralized RM program, the VANCDSP. Second, the study population was largely male and white, which may limit generalizability to other patient populations. Third, although we adjusted for many granular patient- and device-level characteristics, residual confounding is likely given baseline differences in comorbidities and device alert burden between groups. Patients receiving remote-only care had lower comorbidity burden and fewer device alerts; thus, our conclusions may apply primarily to patients with lower risk. Fourth, classification of in-person CIED care was based on CPT codes and VHA stop codes, which identify the occurrence of CIED evaluations but do not provide detail about the clinical context (such as whether the visit was routine or symptom driven or resulted in treatment changes); this limits our ability to fully characterize the intent and impact of in-person evaluations. Fifth, although we assessed MACE outcomes because these are clinical outcomes that are important to patients, other outcomes may be more CIED specific, such as inappropriate ICD therapy and time to intervention after alerts, and should be considered in future studies. Finally, although we had 1-year follow-up data, prospective studies will need to provide longer-term follow-up to ensure the safety and effectiveness of this remote-first care strategy and establish a causal relationship with outcomes if it is to become the standard of care.

## Conclusion

In this large, integrated health care system study, we found that the odds of MACE were similar regardless of whether patients engaged in RM had at least 1 in-person or remote-only evaluations for CIED care. These results support recent professional society consensus recommendations to reduce routine evaluations for CIED care among patients with RM. To implement a remote-first care strategy, systems and device clinics will need to ensure appropriate patient selection, thoughtful program design and resource allocation, and patient adherence to RM through targeted follow-up strategies.

## Disclosures

The authors have no conflicts of interest to disclose.
