# Supplementary material for: Outcomes associated with remote monitoring without in-person evaluations for patients with cardiovascular implantable electronic devices
Source: Heart Rhythm O2. 2025 Aug 7;6(11):1752–60. doi: 10.1016/j.hroo.2025.07.021 (PMC12675077; doi:10.1016/j.hroo.2025.07.021)
Supplement: Supplementary Tables 1-8 [file mmc1.docx]

### Supplementary Data

**Supplemental Table 1**: International Classification of Diseases, Tenth Revision (ICD-10), Current Procedural Terminology (CPT), and Healthcare Common Procedure Coding System (HCPCS) Codes for Any Generator or Lead-Related Procedures used to identify patients who received pacemaker or Implantable Cardioverter-Defibrilator (ICD) care outside the Veterans Health Administration (VHA)

| **Coding System** | **Code** | **Description** |
| --- | --- | --- |
| ICD10 | 02PA0MZ | Removal of cardiac lead from heart, open approach |
| ICD10 | 02PA3MZ | Removal of cardiac lead from heart, percutaneous approach |
| ICD10 | 02PA4MZ | Removal of cardiac lead from heart, percutaneous endoscopic approach |
| ICD10 | 02PAXMZ | Removal of cardiac lead from heart, external approach |
| ICD10 | 02WA0MZ | Revision of cardiac lead in heart, open approach |
| ICD10 | 02WA3MZ | Revision of cardiac lead in heart, percutaneous approach |
| ICD10 | 02WA4MZ | Revision of cardiac lead in heart, percutaneous endoscopic approach |
| ICD10 | 02WAXMZ | Revision of cardiac lead in heart, external approach |
| ICD10 | 02H4[034]JZ | Insertion of pacemaker lead into coronary vein, [open, perc, perc endo] approach |
| ICD10 | 02H6[034]JZ | Insertion of pacemaker lead into right atrium, [open, perc, perc endo] approach |
| ICD10 | 02H7[034]JZ | Insertion of pacemaker lead into left atrium, [open, perc, perc endo] approach |
| ICD10 | 02HK[034]JZ | Insertion of pacemaker lead into right ventricle, [open, perc, perc endo] approach |
| ICD10 | 02HL[034]JZ | Insertion of pacemaker lead into left ventricle, [open, perc, perc endo] |
| ICD10 | 02HN[034]JZ | Insertion of pacemaker lead into pericardium, [open, perc, perc endo] approach |
| ICD10 | 02H4[034]MZ | Insertion of cardiac lead into coronary vein, [open, perc, perc endo] approach |
| ICD10 | 02H6[034]MZ | Insertion of cardiac lead into right atrium, [open, perc, perc endo] approach |
| ICD10 | 02H7[034]MZ | nsertion of cardiac lead into left atrium, [open, perc, perc endo] approach |
| ICD10 | 02HK[034]MZ | Insertion of cardiac lead into right ventricle, [open, perc, perc endo] approach |
| ICD10 | 02HL[034]MZ | Insertion of cardiac lead into left ventricle, [open, perc, perc endo] approach |
| ICD10 | 02HN[034]MZ | Insertion of cardiac lead into pericardium, [open, perc, perc endo] approach |
| ICD10 | 02H4[034]KZ | Insertion of defibrillator lead into coronary vein, [open, percutaneous endoscopic] approach |
| ICD10 | 02H6[034]KZ | Insertion of defibrillator lead into right atrium, [open, percutaneous, percutaneous endoscopic] approach |
| ICD10 | 02H7[034]KZ | Insertion of defibrillator lead into left atrium, [open, percutaneous, percutaneous endoscopic] approach |
| ICD10 | 02HK[034]KZ | Insertion of defibrillator lead into right ventricle, [open, percutaneous, percutaneous endoscopic] approach |
| ICD10 | 02HL[034]KZ | Insertion of defibrillator lead into left ventricle, [open, percutaneous, percutaneous endoscopic] approach |
| ICD10 | 02HN[034]KZ | Insertion of defibrillator lead into pericardium, [open, percutaneous, percutaneous endoscopic] approach |
| ICD10 | 0JWT0PZ | Revision of cardiac rhythm related device in trunk subcutaneous tissue and fascia, open approach |
| ICD10 | 0JWT3PZ | Revision of cardiac rhythm related device in trunk subcutaneous tissue and fascia, percutaneous approach |
| ICD10 | 0JH6[03]4Z | Insert pace, singl cham in chest subcu/fascia, [open, perc] |
| ICD10 | 0JH605Z | Insert pace, singl cham rt respn in chest subcu/fascia, open |
| ICD10 | 0JH6[03]6Z | Insert pace. Dual cham in chest subcu/fascia, [open, perc] |
| ICD10 | 0JH6[03]7Z | Insert card rsync pace puls gen in chest subcu/fascia, [open, perc] |
| ICD10 | 0JH6[03]8Z | Insert of defib gen into chest subcu/fascia, [open approach, perc approach] |
| ICD10 | 0JH6[03]9Z | Insert card rsync defib puls gen in chest subcu/fascia, [open, perc] |
| ICD10 | 0JH6[03]PZ | Insert card rhythm dev in chest subcu/fascia, [open, perc] |
| ICD10 | 0JH808Z | Insertion of defib gen into abd subcu/fascia, open approach |
| ICD10 | 0JH809Z | Insert card rsync defib puls gen in abd subcu/fascia, open |
| ICD10 | 0JPT[03]PZ | Remove card rhythm dev from trunk subcu/fascia, [open, perc] |
| CPT | 33202 | Insertion of epicardial electrodes |
| CPT | 33203 | Insertion of epicardial electrodes |
| CPT | 33206 | Insertion of new or replacement of permanent pacemaker with transvenous electrode(s); atrial |
| CPT | 33207 | Insertion of new or replacement of permanent pacemaker with transvenous electrode(s); ventricular |
| CPT | 33208 | Insertion of new or replacement of permanent pacemaker with transvenous electrode(s); atrial and ventricular |
| CPT | 33212 | Insertion of pacemaker pulse generator only; with existing single lead |
| CPT | 33213 | Insertion of pacemaker pulse generator only; with existing dual leads |
| CPT | 33214 | Upgrade of implanted pacemaker system, conversion of single chamber system to dual chamber system (includes removal of previously placed pulse generator, testing of existing lead, insertion of new lead, insertion of new pulse generator |
| CPT | 33215 | repositioning of previously implanted transvenous pacemaker or implantable defibrillator (right atrial or right ventricular) electrode |
| CPT | 33216 | Insertion of a single transvenous electrode, permanent pacemaker or implantable defibrillator |
| CPT | 33217 | Insertion of 2 transvenous electrodes, permanent pacemaker or implantable defibrillator |
| CPT | 33218 | Repair of single transvenous electrode, permanent pacemaker or implantable defibrillator |
| CPT | 33220 | Repair of 2 transvenous electrodes for permanent pacemaker or implantable defibrillator |
| CPT | 33222 | Relocation of skin pocket for pacemaker |
| CPT | 33223 | Relocation of skin pocket for implantable defibrillator |
| CPT | 33224 | Insertion of pacing electrode, cardiac venous system, for left ventricular pacing, with attachment to previously placed pacemaker or pacing cardioverter-defibrillator pulse generator |
| CPT | 33225 | Insertion of pacing electrode, cardiac venous system, for left ventricular pacing, at time of insertion of implantable defibrillator or pacemaker pulse generator (eg, for upgrade to dual chamber system) (List separately in addition to code for primary procedure) |
| CPT | 33226 | Repositioning of previously implanted cardiac venous system (left ventricular) electrode (including removal, insertion, and/or replacement of existing generator) |
| CPT | 33227 | Removal of permanent pacemaker pulse generator with replacement of pacemaker pulse generator; single lead system |
| CPT | 33228 | Removal of permanent pacemaker pulse generator with replacement of pacemaker pulse generator; dual lead system |
| CPT | 33229 | Removal of permanent pacemaker pulse generator with replacement of pacemaker pulse generator; multiple lead system |
| CPT | 33230 | Insertion of implantable defibrillator pulse generator only; with existing dual leads |
| CPT | 33231 | Insertion of implantable defibrillator pulse generator only; with existing multiple leads |
| CPT | 33233 | Removal of permanent pacemaker pulse generator only |
| CPT | 33234 | Removal of transvenous pacemaker electrode(s); single lead system, atrial or ventricular |
| CPT | 33235 | Removal of transvenous pacemaker electrode(s); dual lead system |
| CPT | 33236 | Removal of permanent epicardial pacemaker and electrodes by thoracotomy; single lead system, atrial or ventricular |
| CPT | 33237 | Removal of permanent epicardial pacemaker and electrodes by thoracotomy; dual lead system |
| CPT | 33238 | Removal of permanent transvenous electrode(s) by thoracotomy |
| CPT | 33240 | insertion of implantable defibrillator pulse generator only; with existing single lead |
| CPT | 33241 | removal of implantable defibrillator pulse generator only |
| CPT | 33243 | Removal of single or dual chamber implantable defibrillator electrode(s); by thoracotomy |
| CPT | 33244 | Removal of single or dual chamber implantable defibrillator electrode(s); by transvenous extraction |
| CPT | 33249 | Insertion or replacement of permanent implantable defibrillator system, with transvenous lead(s), single or dual chamber |
| CPT | 33262 | Removal of implantable defibrillator pulse generator with replacement of implantable defibrillator pulse generator; single lead system |
| CPT | 33263 | Removal of implantable defibrillator pulse generator with replacement of implantable defibrillator pulse generator; dual lead system |
| CPT | 33264 | Removal of implantable defibrillator pulse generator with replacement of implantable defibrillator pulse generator; multiple lead system |
| CPT | 33270 | Insertion or replacement of permanent subcutaneous implantable defibrillator system, with subcutaneous electrode, including defibrillation thresh old evaluation, induction of arrhythmia, evaluation of sensing for arrhythmia termination, and programming or reprogramming of sensing or therapeutic parameters, when performed |
| CPT | 33271 | Insertion of subcutaneous implantable defibrillator electrode |
| CPT | 33272 | Removal of subcutaneous implantable defibrillator electrode |
| CPT | 33273 | Repositioning of previously implanted subcutaneous implantable defibrillator electrode |
| HCPCS | G0448 | Insertion or replacement of a permanent pacing cardioverter-defibrillator system with transvenous lead(s), single or dual chamber with insertion of pacing electrode, cardiac venous system, for left ventricular pacing |
| HCPCS | C1721 | Cardioverter-defibrillator, dual chamber (implantable) |
| HCPCS | C1722 | Cardioverter-defibrillator, single chamber (implantable) |
| HCPCS | C1777 | Lead, cardioverter-defibrillator, endocardial single coil (implantable) |
| HCPCS | C1779 | Lead, pacemaker, transvenous vdd single pass |
| HCPCS | C1785 | Pacemaker, dual chamber, rate-responsive (implantable) |
| HCPCS | C1786 | Pacemaker, single chamber, rate-responsive (implantable) |
| HCPCS | C1882 | Cardioverter-defibrillator, other than single or dual chamber (implantable) |
| HCPCS | C1895 | Lead, cardioverter-defibrillator, endocardial dual coil (implantable) |
| HCPCS | C1896 | Lead, cardioverter-defibrillator, other than endocardial single or dual coil (implantable) |
| HCPCS | C1898 | Lead, pacemaker, other than transvenous vdd single pass |
| HCPCS | C1899 | Lead, pacemaker/cardioverter-defibrillator combination (implantable) |
| HCPCS | C1900 | Lead, left ventricular coronary venous system |
| HCPCS | C2621 | Pacemaker, other than single or dual chamber (implantable) |

**Supplemental Table 2:** Current Procedural Terminology **(**CPT) Codes for In-person and Remote Implantable Cardioverter-Defibrilator (ICD) or Pacemaker Evaluation

| **CPT code** | **Description** |
| --- | --- |
| IN-PERSON EVALUATION | |
| 93260 | Programming device evaluation (in person) with iterative adjustment of the implantable device to test the function of the device and select optimal permanent programmed values with analysis, review and report by a physician or other qualified health care professional; implantable subcutaneous lead defibrillator system |
| 93261 | Interrogation device evaluation (in person) with analysis, review and report by a physician or other qualified health care professional, includes connection, recording and disconnection per patient encounter; implantable subcutaneous lead defibrillator system |
| 93279 | Programming device evaluation (in person) with iterative adjustment of the implantable device to test the function of the device and select optimal permanent programmed values with analysis, review and report by a physician or other qualified health care professional; single lead pacemaker system or leadless pacemaker system in one cardiac chamber |
| 93280 | Programming device evaluation (in person) with iterative adjustment of the implantable device to test the function of the device and select optimal permanent programmed values with analysis, review and report by a physician or other qualified health care professional; dual lead pacemaker system |
| 93281 | Programming device evaluation (in person) with iterative adjustment of the implantable device to test the function of the device and select optimal permanent programmed values with analysis, review and report by a physician or other qualified health care professional; multiple lead pacemaker system |
| 93282 | Programming device evaluation (in person) with iterative adjustment of the implantable device to test the function of the device and select optimal permanent programmed values with analysis, review and report by a physician or other qualified health care professional; single lead transvenous implantable defibrillator system |
| 93283 | Programming device evaluation (in person) with iterative adjustment of the implantable device to test the function of the device and select optimal permanent programmed values with analysis, review and report by a physician or other qualified health care professional; dual lead transvenous implantable defibrillator system |
| 93284 | Programming device evaluation (in person) with iterative adjustment of the implantable device to test the function of the device and select optimal permanent programmed values with analysis, review and report by a physician or other qualified health care professional; multiple lead transvenous implantable defibrillator system |
| 93286 | Peri-procedural device evaluation (in person) and programming of device system parameters before or after a surgery, procedure, or test with analysis, review and report by a physician or other qualified health care professional; single, dual, or multiple lead pacemaker system or leadless pacemaker system |
| 93287 | Peri-procedural device evaluation (in person) and programming of device system parameters before or after a surgery, procedure, or test with analysis, review and report by a physician or other qualified health care professional; single, dual, or multiple lead implantable defibrillator system |
| 93288 | Interrogation device evaluation (in person) with analysis, review, and report by a physician or other qualified healthcare professional, includes connection, recording, and disconnection per patient encounter; single, dual, or multiple lead pacemaker system or leadless pacemaker system |
| 93289 | Interrogation device evaluation (in person) with analysis, review and report by a physician or other qualified health care professional, includes connection, recording and disconnection per patient encounter; single, dual, or multiple lead transvenous implantable defibrillator system, including analysis of heart rhythm derived data elements (For monitoring physiologic cardiovascular data elements derived from an implantable defibrillator, use 93290) |
| REMOTE EVALUATION | |
| 93294 | Interrogation device evaluation(s) (remote), up to 90 days; single, dual, or multiple lead pacemaker system, or leadless pacemaker system with interim analysis, review(s) and report(s) by a physician or other qualified health care professional |
| 93295 | Interrogation device evaluation(s) (remote), up to 90 days; single, dual, or multiple lead implantable defibrillator system with interim analysis, review(s) and report(s) by a physician or other qualified health care professional (For remote monitoring of physiologic cardiovascular data elements derived from an ICD, use 93297) |
| 93296 | Interrogation device evaluation(s) (remote), up to 90 days; single, dual, or multiple lead pacemaker system, leadless pacemaker system, or implantable defibrillator system, remote data acquisition(s), receipt of transmissions and technician review, technical support and distribution of results |

**Supplemental Table 3:** Healthcare Common Procedure Coding System (HCPCS) Codes for Hospice

| **Coding System** | **Code** | **Description** |
| --- | --- | --- |
| HCPCS | Q5003 | Hospice care provided in nursing long term care facility (LTC) or non-skilled nursing facility (NF) |
| HCPCS | Q5004 | Hospice care provided in skilled nursing facility (SNF) |
| HCPCS | Q5005 | Hospice care provided in inpatient hospital |
| HCPCS | Q5006 | Hospice care provided in inpatient hospice facility |
| HCPCS | Q5007 | Hospice care provided in long term care facility |
| HCPCS | Q5008 | Hospice care provided in inpatient psychiatric facility |
| HCPCS | Q5010 | Hospice home care provided in a hospice facility |
| HCPCS | T2042 | Hospice routine home care; per diem |
| HCPCS | T2043 | Hospice continuous home care; per hour |
| HCPCS | T2044 | Hospice inpatient respite care; per diem |
| HCPCS | T2045 | Hospice general inpatient care; per diem |
| HCPCS | T2046 | Hospice long term care, room and board only; per diem |

**Supplemental Table 4:** International Classification of Diseases, Tenth Revision (ICD-10) Codes for Cardiac Hospitalization

| **ICD-10 Code** | **Code Description** |
| --- | --- |
| I44.0 | Atrioventricular block, first degree |
| I44.1 | Atrioventricular block, second degree |
| I44.2 | Atrioventricular block, complete |
| I44.30 | Unspecified atrioventricular block |
| I44.39 | Other atrioventricular block |
| I44.4 | Left anterior fascicular block |
| I44.5 | Left posterior fascicular block |
| I44.60 | Unspecified fascicular block |
| I44.69 | Other fascicular block |
| I44.7 | Left bundle-branch block, unspecified |
| I45.0 | Right fascicular block |
| I45.10 | Unspecified right bundle-branch block |
| I45.19 | Other right bundle-branch block |
| I45.2 | Bifascicular block |
| I45.3 | Trifascicular block |
| I45.4 | Nonspecific intraventricular block |
| I45.5 | Other specified heart block |
| I45.6 | Pre-excitation syndrome |
| I45.81 | Long QT syndrome |
| I45.89 | Other specified conduction disorders |
| I45.9 | Conduction disorder, unspecified |
| I47.0 | Re-entry ventricular arrhythmia |
| I47.1 | Supraventricular tachycardia |
| I47.2 | Ventricular tachycardia |
| I47.9 | Paroxysmal tachycardia, unspecified |
| I48.0 | Paroxysmal atrial fibrillation |
| I48.11 | Longstanding persistent atrial fibrillation |
| I48.19 | Other persistent atrial fibrillation |
| I48.20 | Chronic atrial fibrillation, unspecified |
| I48.21 | Permanent atrial fibrillation |
| I48.3 | Typical atrial flutter |
| I48.4 | Atypical atrial flutter |
| I48.91 | Unspecified atrial fibrillation |
| I48.92 | Unspecified atrial flutter |
| I49.01 | Ventricular fibrillation |
| I49.02 | Ventricular flutter |
| I49.1 | Atrial premature depolarization |
| I49.2 | Junctional premature depolarization |
| I49.3 | Ventricular premature depolarization |
| I49.40 | Unspecified premature depolarization |
| I49.49 | Other premature depolarization |
| I49.5 | Sick sinus syndrome |
| I49.8 | Other specified cardiac arrhythmias |
| I49.9 | Cardiac arrhythmia, unspecified |
| I20.0 | Unstable angina |
| I20.1 | Angina pectoris with documented spasm |
| I20.8 | Other forms of angina pectoris |
| I20.9 | Angina pectoris, unspecified |
| I21.01 | ST elevation (STEMI) myocardial infarction involving left main coronary artery |
| I21.02 | ST elevation (STEMI) myocardial infarction involving left anterior descending coronary artery |
| I21.09 | ST elevation (STEMI) myocardial infarction involving other coronary artery of anterior wall |
| I21.11 | ST elevation (STEMI) myocardial infarction involving right coronary artery |
| I21.19 | ST elevation (STEMI) myocardial infarction involving other coronary artery of inferior wall |
| I21.21 | ST elevation (STEMI) myocardial infarction involving left circumflex coronary artery |
| I21.29 | ST elevation (STEMI) myocardial infarction involving other sites |
| I21.3 | ST elevation (STEMI) myocardial infarction of unspecified site |
| I21.4 | Non-ST elevation (NSTEMI) myocardial infarction |
| I21.9 | Acute myocardial infarction, unspecified |
| I21.A9 | Other myocardial infarction type |
| I22.0 | Subsequent ST elevation (STEMI) myocardial infarction of anterior wall |
| I22.1 | Subsequent ST elevation (STEMI) myocardial infarction of inferior wall |
| I22.2 | Subsequent non-ST elevation (NSTEMI) myocardial infarction |
| I22.8 | Subsequent ST elevation (STEMI) myocardial infarction of other sites |
| I22.9 | Subsequent ST elevation (STEMI) myocardial infarction of unspecified site |
| I23.0 | Hemopericardium as current complication following acute myocardial infarction |
| I23.1 | Atrial septal defect as current complication following acute myocardial infarction |
| I23.2 | Ventricular septal defect as current complication following acute myocardial infarction |
| I23.3 | Rupture of cardiac wall without hemopericardium as current complication following acute myocardial infarction |
| I23.4 | Rupture of chordae tendineae as current complication following acute myocardial infarction |
| I23.5 | Rupture of papillary muscle as current complication following acute myocardial infarction |
| I23.6 | Thrombosis of atrium, auricular appendage, and ventricle as current complications following acute myocardial infarction |
| I23.7 | Postinfarction angina |
| I23.8 | Other current complications following acute myocardial infarction |
| I24.0 | Acute coronary thrombosis not resulting in myocardial infarction |
| I24.1 | Dressler's syndrome |
| I24.8 | Other forms of acute ischemic heart disease |
| I24.9 | Acute ischemic heart disease, unspecified |
| I25.10 | Atherosclerotic heart disease of native coronary artery without angina pectoris |
| I25.110 | Atherosclerotic heart disease of native coronary artery with unstable angina pectoris |
| I25.111 | Atherosclerotic heart disease of native coronary artery with angina pectoris with documented spasm |
| I25.118 | Atherosclerotic heart disease of native coronary artery with other forms of angina pectoris |
| I25.119 | Atherosclerotic heart disease of native coronary artery with unspecified angina pectoris |
| I25.42 | Coronary artery dissection |
| I25.5 | Ischemic cardiomyopathy |
| I25.6 | Silent myocardial ischemia |
| I25.700 | Atherosclerosis of coronary artery bypass graft(s), unspecified, with unstable angina pectoris |
| I25.701 | Atherosclerosis of coronary artery bypass graft(s), unspecified, with angina pectoris with documented spasm |
| I25.708 | Atherosclerosis of coronary artery bypass graft(s), unspecified, with other forms of angina pectoris |
| I25.709 | Atherosclerosis of coronary artery bypass graft(s), unspecified, with unspecified angina pectoris |
| I25.710 | Atherosclerosis of autologous vein coronary artery bypass graft(s) with unstable angina pectoris |
| I25.711 | Atherosclerosis of autologous vein coronary artery bypass graft(s) with angina pectoris with documented spasm |
| I25.718 | Atherosclerosis of autologous vein coronary artery bypass graft(s) with other forms of angina pectoris |
| I25.719 | Atherosclerosis of autologous vein coronary artery bypass graft(s) with unspecified angina pectoris |
| I25.720 | Atherosclerosis of autologous artery coronary artery bypass graft(s) with unstable angina pectoris |
| I25.721 | Atherosclerosis of autologous artery coronary artery bypass graft(s) with angina pectoris with documented spasm |
| I25.728 | Atherosclerosis of autologous artery coronary artery bypass graft(s) with other forms of angina pectoris |
| I25.729 | Atherosclerosis of autologous artery coronary artery bypass graft(s) with unspecified angina pectoris |
| I25.730 | Atherosclerosis of nonautologous biological coronary artery bypass graft(s) with unstable angina pectoris |
| I25.731 | Atherosclerosis of nonautologous biological coronary artery bypass graft(s) with angina pectoris with documented spasm |
| I25.738 | Atherosclerosis of nonautologous biological coronary artery bypass graft(s) with other forms of angina pectoris |
| I25.739 | Atherosclerosis of nonautologous biological coronary artery bypass graft(s) with unspecified angina pectoris |
| I25.750 | Atherosclerosis of native coronary artery of transplanted heart with unstable angina |
| I25.751 | Atherosclerosis of native coronary artery of transplanted heart with angina pectoris with documented spasm |
| I25.758 | Atherosclerosis of native coronary artery of transplanted heart with other forms of angina pectoris |
| I25.759 | Atherosclerosis of native coronary artery of transplanted heart with unspecified angina pectoris |
| I25.760 | Atherosclerosis of bypass graft of coronary artery of transplanted heart with unstable angina |
| I25.761 | Atherosclerosis of bypass graft of coronary artery of transplanted heart with angina pectoris with documented spasm |
| I25.768 | Atherosclerosis of bypass graft of coronary artery of transplanted heart with other forms of angina pectoris |
| I25.769 | Atherosclerosis of bypass graft of coronary artery of transplanted heart with unspecified angina pectoris |
| I25.790 | Atherosclerosis of other coronary artery bypass graft(s) with unstable angina pectoris |
| I25.791 | Atherosclerosis of other coronary artery bypass graft(s) with angina pectoris with documented spasm |
| I25.798 | Atherosclerosis of other coronary artery bypass graft(s) with other forms of angina pectoris |
| I25.799 | Atherosclerosis of other coronary artery bypass graft(s) with unspecified angina pectoris |
| I25.810 | Atherosclerosis of coronary artery bypass graft(s) without angina pectoris |
| I25.811 | Atherosclerosis of native coronary artery of transplanted heart without angina pectoris |
| I25.812 | Atherosclerosis of bypass graft of coronary artery of transplanted heart without angina pectoris |
| I25.82 | Chronic total occlusion of coronary artery |
| I25.83 | Coronary atherosclerosis due to lipid rich plaque |
| I25.84 | Coronary atherosclerosis due to calcified coronary lesion |
| I25.89 | Other forms of chronic ischemic heart disease |
| I25.9 | Chronic ischemic heart disease, unspecified |
| I46.2 | Cardiac arrest due to underlying cardiac condition |
| I46.8 | Cardiac arrest due to other underlying condition |
| I46.9 | Cardiac arrest, cause unspecified |
| I50.1 | Left ventricular failure, unspecified |
| I50.20 | Unspecified systolic (congestive) heart failure |
| I50.21 | Acute systolic (congestive) heart failure |
| I50.22 | Chronic systolic (congestive) heart failure |
| I50.23 | Acute on chronic systolic (congestive) heart failure |
| I50.30 | Unspecified diastolic (congestive) heart failure |
| I50.31 | Acute diastolic (congestive) heart failure |
| I50.32 | Chronic diastolic (congestive) heart failure |
| I50.33 | Acute on chronic diastolic (congestive) heart failure |
| I50.40 | Unspecified combined systolic (congestive) and diastolic (congestive) heart failure |
| I50.41 | Acute combined systolic (congestive) and diastolic (congestive) heart failure |
| I50.42 | Chronic combined systolic (congestive) and diastolic (congestive) heart failure |
| I50.43 | Acute on chronic combined systolic (congestive) and diastolic (congestive) heart failure |
| I50.810 | Right heart failure, unspecified |
| I50.811 | Acute right heart failure |
| I50.812 | Chronic right heart failure |
| I50.813 | Acute on chronic right heart failure |
| I50.814 | Right heart failure due to left heart failure |
| I50.82 | Biventricular heart failure |
| I50.83 | High output heart failure |
| I50.84 | End stage heart failure |
| I50.89 | Other heart failure |
| I50.9 | Heart failure, unspecified |
| R55. | Syncope and collapse |

**Supplemental Table 5:** International Classification of Diseases, Tenth Revision (ICD-10) Codes for Comorbidities

| **Comorbidities** | **ICD-10 codes** |
| --- | --- |
| Cancer (excluding non-melanoma skin cancer) | C00.x-C96.x, C7A |
| Chronic kidney disease | A18.11, A52.75, B52.0, C64.1, C64.2, C64.9, C68.9, D30.00, D30.01, D30.02, D41.00, D41.01, D41.02, D41.10, D41.11, D41.12, D41.20, D41.21, D41.22, D59.3, E08.21, E08.22, E08.29, E08.65, E09.21, E09.22, E09.29, E10.21, E10.22, E10.29, E10.65, E11.21, E11.22, E11.29, E11.65, E13.21, E13.22, E13.29, E74.8, I12.0, I12.9, I13.0, I13.10, I13.11, I13.2, I70.1, I72.2, K76.7, M10.30, M10.311, M10.312, M10.319, M10.321, M10.322, M10.329, M10.331, M10.332, M10.339, M10.341, M10.342, M10.349, M10.351, M10.352, M10.359, M10.361, M10.362, M10.369, M10.371, M10.372, M10.379, M10.38, M10.39, M32.14, M32.15, M35.04, N00.0, N00.1, N00.2, N00.3, N00.4, N00.5, N00.6, N00.7, N00.8, N00.9, N01.0, N01.1, N01.2, N01.3, N01.4, N01.5, N01.6, N01.7, N01.8, N01.9, N02.0, N02.1, N02.2, N02.3, N02.4, N02.5, N02.6, N02.7, N02.8, N02.9, N03.0, N03.1, N03.2, N03.3, N03.4, N03.5, N03.6, N03.7, N03.8, N03.9, N04.0, N04.1, N04.2, N04.3, N04.4, N04.5, N04.6, N04.7, N04.8, N04.9, N05.0, N05.1, N05.2, N05.3, N05.4, N05.5, N05.6, N05.7, N05.8, N05.9, N06.0, N06.1, N06.2, N06.3, N06.4, N06.5, N06.6, N06.7, N06.8, N06.9, N07.0, N07.1, N07.2, N07.3, N07.4, N07.5, N07.6, N07.7, N07.8, N07.9, N08, N13.1, N13.2, N13.30, N13.39, N14.0, N14.1, N14.2, N14.3, N14.4, N15.0, N15.8, N15.9, N16, N17.0, N17.1, N17.2, N17.8, N17.9, N18.1, N18.2, N18.3, N18.4, N18.5, N18.6, N18.9, N19, N25.0, N25.1, N25.81, N25.89, N25.9, N26.1, N26.9, Q61.02, Q61.11, Q61.19, Q61.2, Q61.3, Q61.4, Q61.5, Q61.8, Q62.0, Q62.2, Q62.10, Q62.11, Q62.12, Q62.31, Q62.32, Q62.39, R94.4 |
| End-stage renal disease | N18.5, N18.6, I12.0, I13.11, I13.2, N18.5, N18.6, Z49.x, Z94.0, Z99.2 |
| Chronic obstructive pulmonary disease | J40, J41.0, J41.1, J41.8, J42, J43.0, J43.1, J43.2, J43.8, J43.9, J44.0, J44.1, J44.9, J47.0, J47.1, J47.9 |
| Diabetes mellitus | E08.00, E08.01, E08.10, E08.11, E08.21, E08.22, E08.29, E08.311, E08.319, E08.321, E08.3211, E08.3212, E08.3213, E08.3219, E08.329, E08.3291, E08.3292, E08.3293, E08.3299, E08.331, E08.3311, E08.3312, E08.3313, E08.3319, E08.339, E08.3391, E08.3392, E08.3393, E08.3399, E08.341, E08.3411, E08.3412, E08.3413, E08.3419, E08.349, E08.3491, E08.3492, E08.3493, E08.3499, E08.351, E08.3511, E08.3512, E08.3513, E08.3519, E08.3521, E08.3522, E08.3523, E08.3529, E08.3531, E08.3532, E08.3533, E08.3539, E08.3541, E08.3542, E08.3543, E08.3549, E08.3551, E08.3552, E08.3553, E08.3559, E08.359, E08.3591, E08.3592, E08.3593, E08.3599, E08.36, E08.37X1, E08.37X2, E08.37X3, E08.37X9, E08.39, E08.40, E08.41, E08.42, E08.43, E08.44, E08.49, E08.51, E08.52, E08.59, E08.610, E08.618, E08.620, E08.621, E08.622, E08.628, E08.630, E08.638, E08.641, E08.649, E08.65, E08.69, E08.8, E08.9, E09.00, E09.01, E09.10, E09.11, E09.21, E09.22, E09.29, E09.311, E09.319, E09.321, E09.3211, E09.3212, E09.3213, E09.3219, E09.329, E09.3291, E09.3292, E09.3293, E09.3299, E09.331, E09.3311, E09.3312, E09.3313, E09.3319, E09.339, E09.3391, E09.3392, E09.3393, E09.3399, E09.341, E09.3411, E09.3412, E09.3413, E09.3419, E09.349, E09.3491, E09.3492, E09.3493, E09.3499, E09.351, E09.3511, E09.3512, E09.3513, E09.3519, E09.3521, E09.3522, E09.3523, E09.3529, E09.3531, E09.3532, E09.3533, E09.3539, E09.3541, E09.3542, E09.3543, E09.3549, E09.3551, E09.3552, E09.3553, E09.3559, E09.359, E09.3591, E09.3592, E09.3593, E09.3599, E09.36, E09.37X1, E09.37X2, E09.37X3, E09.37X9, E09.39, E09.40, E09.41, E09.42, E09.43, E09.44, E09.49, E09.51, E09.52, E09.59, E09.610, E09.618, E09.620, E09.621, E09.622, E09.628, E09.630, E09.638, E09.641, E09.649, E09.65, E09.69, E09.8, E09.9, E10.10, E10.11, E10.21, E10.22, E10.29, E10.311, E10.319, E10.321, E10.3211, E10.3212, E10.3213, E10.3219, E10.329, E10.3291, E10.3292, E10.3293, E10.3299, E10.331, E10.3311, E10.3312, E10.3313, E10.3319, E10.339, E10.3391, E10.3392, E10.3393, E10.3399, E10.341, E10.3411, E10.3412, E10.3413, E10.3419, E10.349, E10.3491, E10.3492, E10.3493, E10.3499, E10.351, E10.3511, E10.3512, E10.3513, E10.3519, E10.359, E10.36, E10.37X1, E10.37X2, E10.37X3, E10.37X9, E10.39, E10.40, E10.41, E10.42, E10.43, E10.44, E10.49, E10.51, E10.52, E10.59, E10.610, E10.618, E10.620, E10.621, E10.622, E10.628, E10.630, E10.638, E10.641, E10.649, E10.65, E10.69, E10.8, E10.9, E11.00, E11.01, E11.10, E11.11, E11.21, E11.22, E11.29, E11.311, E11.319, E11.321, E11.3211, E11.3212, E11.3213, E11.3219, E11.329, E11.3291, E11.3292, E11.3293, E11.3299, E11.331, E11.3311, E11.3312, E11.3313, E11.3319, E11.339, E11.3391, E11.3392, E11.3393, E11.3399, E11.341, E11.3411, E11.3412, E11.3413, E11.3419, E11.349, E11.3491, E11.3492, E11.3493, E11.3499, E11.351, E11.3511, E11.3512, E11.3513, E11.3519, E11.3521, E11.3522, E11.3523, E11.3529, E11.3531, E11.3532, E11.3533, E11.3539, E11.3541, E11.3542, E11.3543, E11.3549, E11.3551, E11.3552, E11.3553, E11.3559, E11.359, E11.3591, E11.3592, E11.3593, E11.3599, E11.36, E11.37X1, E11.37X2, E11.37X3, E11.37X9, E11.39, E11.40, E11.41, E11.42, E11.43, E11.44, E11.49, E11.51, E11.52, E11.59, E11.610, E11.618, E11.620, E11.621, E11.622, E11.628, E11.630, E11.638, E11.641, E11.649, E11.65, E11.69, E11.8, E11.9, E13.00, E13.01, E13.10, E13.11, E13.21, E13.22, E13.29, E13.311, E13.319, E13.321, E13.3211, E13.3212, E13.3213, E13.3219, E13.329, E13.3291, E13.3292, E13.3293, E13.3299, E13.331, E13.3311, E13.3312, E13.3313, E13.3319, E13.339, E13.3391, E13.3392, E13.3393, E13.3399, E13.341, E13.3411, E13.3412, E13.3413, E13.3419, E13.349, E13.3491, E13.3492, E13.3493, E13.3499, E13.351, E13.3511, E13.3512, E13.3513, E13.3519, E13.3521, E13.3522, E13.3523, E13.3529, E13.3531, E13.3532, E13.3533, E13.3539, E13.3541, E13.3542, E13.3543, E13.3549, E13.3551, E13.3552, E13.3553, E13.3559, E13.359, E13.36, E13.39, E13.40, E13.41, E13.42, E13.43, E13.44, E13.49, E13.51, E13.52, E13.59, E13.610, E13.618, E13.620, E13.621, E13.622, E13.628, E13.630, E13.638, E13.641, E13.649, E13.65, E13.69, E13.8, E13.9 |
| Dyslipidemia | E78.0, E78.00, E78.01, E78.1, E78.2, E78.3, E78.4, E78.41, E78.49, E78.5 |
| Hypertension | H35.031, H35.032, H35.033, H35.039, I10, I11.0, I11.9, I12.0, I12.9, I13.0, I13.10, I13.11, I13.2, I15.0, I15.1, I15.2, I15.8, I15.9, I67.4, N26.2 |
| Tobacco use | F17.200, F17.201, F17.203, F17.208, F17.209, F17.210, F17.211, F17.213, F17.218, F17.219, F17.220, F17.221, F17.223, F17.228, F17.229, F17.290, F17.291, F17.293, F17.298, F17.299, O99.330, O99.331, O99.332, O99.333, O99.334, O99.335, T65.211A, T65.212A, T65.213A, T65.214A, T65.221A, T65.222A, T65.223A, T65.224A, T65.291A, T65.292A, T65.293A, T65.294A, Z72.0 |
| Alcohol use | F10.10, F10.120, F10.121, F10.129, F10.14, F10.150, F10.151, F10.159, F10.180, F10.181, F10.182, F10.188, F10.19, F10.20, F10.220, F10.221, F10.229, F10.230, F10.231, F10.232, F10.239, F10.24, F10.250, F10.251, F10.259, F10.26, F10.27, F10.280, F10.281, F10.282, F10.288, F10.29, F10.920, F10.921, F10.929, F10.94, F10.950, F10.951, F10.959, F10.96, F10.97, F10.980, F10.981, F10.982, F10.988, F10.99, G62.1, I42.6, K29.20, K29.21, K70.0, K70.10, K70.11, K70.2, K70.30, K70.31, K70.40, K70.41, K70.9, P04.3, Q86.0, T51.0X1A, T51.0X2A, T51.0X3A, T51.0X4A, Z71.41, Z71.42 |
| Ischemic heart disease | I20.0, I20.1, I20.8, I20.9, I21.01, I21.02, I21.09, I21.11, I21.19, I21.21, I21.29, I21.3, I21.4, I21.A1, I21.A9, I22.0, I22.1, I22.2, I22.8, I22.9, I23.0, I23.1, I23.2, I23.3, I23.4, I23.5, I23.6, I23.7, I23.8, I24.0, I24.1, I24.8, I24.9, I25.10, I25.110, I25.111, I25.118, I25.119, I25.2, I25.3, I25.41, I25.42, I25.5, I25.6, I25.700, I25.701, I25.708, I25.709, I25.710, I25.711, I25.718, I25.719, I25.720, I25.721, I25.728, I25.729, I25.730, I25.731, I25.738, I25.739, I25.750, I25.751, I25.758, I25.759, I25.760, I25.761, I25.768, I25.769, I25.790, I25.791, I25.798, I25.799, I25.810, I25.811, I25.812, I25.82, I25.83, I25.84, I25.89, I25.9 |
| Prior myocardial infarction | I21.x, I22.x, I23.x, I25.2 |
| Prior ventricular tachycardia | I47.2 |
| Valvular heart disease | I05.x, I06.x, I07.x, I08.x, I33.x, I34.x, I35.x, I36.x, I37.x, I38.x, I39.x |
| Atrial fibrillation | I48.0, I48.1, I48.11, I48.19, I48.2, I48.20, I48.21, I48.91 |
| Stroke or transient ischemic attack | G45.0, G45.1, G45.2, G45.8, G45.9, G46.0, G46.1, G46.2, G46.3, G46.4, G46.5, G46.6, G46.7, G46.8, G97.31, G97.32, I60.00, I60.01, I60.02, I60.10, I60.11, I60.12, I60.20, I60.21, I60.22, I60.30, I60.31, I60.32, I60.4, I60.50, I60.51, I60.52, I60.6, I60.7, I60.8, I60.9, I61.0, I61.1, I61.2, I61.3, I61.4, I61.5, I61.6, I61.8, I61.9, I63.00, I63.02, I63.011, I63.012, I63.013, I63.019, I63.02, I63.031, I63.032, I63.039, I63.09, I63.10, I63.111, I63.112, I63.119, I63.12, I63.131, I63.132, I63.139, I63.19, I63.20, I63.211, I63.212, I63.213, I63.219, I63.22, I63.231, I63.232, I63.233, I63.239, I63.29, I63.30, I63.311, I63.312, I63.313, I63.319, I63.321, I63.322, I63.323, I63.329, I63.331, I63.332, I63.333, I63.339, I63.341, I63.342, I63.343, I63.349, I63.39, I63.40, I63.411, I63.412, I63.413, I63.419, I63.421, I63.422, I63.423, I63.429, I63.431, I63.432, I63.433, I63.439, I63.441, I63.442, I63.443, I63.449, I63.49, I63.50, I63.511, I63.512, I63.513, I63.519, I63.521, I63.522, I63.523, I63.529, I63.531, I63.532, I63.533, I63.539, I63.541, I63.542, I63.543, I63.549, I63.59, I63.6, I63.8, I63.9, I66.01, I66.02, I66.03, I66.09, I66.11, I66.12, I66.13, I66.19, I66.21, I66.22, I66.23, I66.29, I66.3, I66.8, I66.9, I67.841, I67.848, I67.89, I97.810, I97.811, I97.820, I97.821 |
| Peripheral vascular disease | E08.51, E08.52, E09.51, E09.52, E10.51, E10.52, E11.51, E11.52, E13.51, E13.52, I70.0, I70.1, I70.201, I70.202, I70.203, I70.208, I70.209, I70.211, I70.212, I70.213, I70.218, I70.219, I70.221, I70.222, I70.223, I70.228, I70.229, I70.231, I70.232, I70.233, I70.234, I70.235, I70.238, I70.239, I70.241, I70.242, I70.243, I70.244, I70.245, I70.248, I70.249, I70.25, I70.291, I70.292, I70.293, I70.298, I70.299, I70.92, I73.81, I73.89, I73.9, I79.1, I79.8 |
| Heart failure | I09.81, I11.0, I13.0, I13.2, I50.1, I50.20, I50.21, I50.22, I50.23, I50.30, I50.31, I50.32, I50.33, I50.40, I50.41, I50.42, I50.43, I50.810, I50.811, I50.812, I50.813, I50.814, I50.82, I50.83, I50.84, I50.89, I50.9, I50.2x, I50.4x |
| Heart transplant recipient | T86.2-T86.298, Z48.21, Z94.1, Z95.812 |
| Interstitial lung disease | J84-J84.178 |

**Supplemental Table 6:** International Classification of Diseases (ICD)-10, Current Procedural Terminology (CPT), and Healthcare Common Procedure Coding System (HCPCS) Codes for percutaneous coronary intervention, coronary artery bypass graft surgery, and left ventricular assist devices

| **Procedure** | **ICD 10** | **CPT** | **HCPCS** |
| --- | --- | --- | --- |
| Percutaneous coronary intervention | 02703ZZ, 02704ZZ, 02713ZZ, 02714ZZ, 02723ZZ, 02724ZZ, 02733ZZ, 02734ZZ, 02C00XX, 02C03ZZ, 02C13ZZ, 02C14ZZ, 02C23ZZ, 02C24ZZ, 02C33ZZ, 02C34ZZ, 3E07317, 3E073PZ | 92920, 92921, 92924, 92925, 92928, 92929, 92933, 92934, 92937, 92938, 92941, 92943, 92944, 92973, 92974, 92975, 92977, 92980, 92981, 92982, 92984, 92995, 92996 | C9600, C9601, C9602, C9603, C9604, C9605, C9606, C9607, C9608, G0290, G0291 |
| Coronary artery bypass graft surgery | 02100XX, 02110XX, 02120XX, 02130XX | 33510, 33511, 33512, 33513, 33514, 33516, 33517. 33518, 33519, 33521, 33522, 33523, 33530, 33533, 33534, 33535, 33536, 33572, 35600 | S2205, S2206, S2207, S2208, S2209 |
| Left ventricular assist device | 02HA0QZ, 02PA0QZ, 02WA0QZ | 33979, 33980, 33982, 33983 | Q0508, Q0509 |

**Supplemental Table 7:** Healthcare Common Procedure Coding System (HCPCS) and Veterans Health Administration (VHA) Stop Codes for Long-term Care Utilization

| **Coding System** | **Code** | **Description** |
| --- | --- | --- |
| HCPCS | 99304 | Initial nursing facility care, per day, for the evaluation and management of a patient, which requires these 3 key components: a detailed or comprehensive history; a detailed or comprehensive examination; and medical decision making that is straightforward or of low complexity. Counseling and/or coordination of care with other physicians, other qualified health care professionals, or agencies are provided consistent with the nature of the problem(s) and the patient's and/or family's needs. Usually, the problem(s) requiring admission are of low severity. Typically, 25 minutes are spent at the bedside and on the patient's facility floor or unit. |
| HCPCS | 99305 | Initial nursing facility care, per day, for the evaluation and management of a patient, which requires these 3 key components: a comprehensive history; a comprehensive examination; and medical decision making of moderate complexity. Counseling and/or coordination of care with other physicians, other qualified health care professionals, or agencies are provided consistent with the nature of the problem(s) and the patient's and/or family's needs. Usually, the problem(s) requiring admission are of moderate severity. Typically, 35 minutes are spent at the bedside and on the patient's facility floor or unit. |
| HCPCS | 99306 | Initial nursing facility care, per day, for the evaluation and management of a patient, which requires these 3 key components: a comprehensive history; a comprehensive examination; and medical decision making of high complexity. Counseling and/or coordination of care with other physicians, other qualified health care professionals, or agencies are provided consistent with the nature of the problem(s) and the patient's and/or family's needs. Usually, the problem(s) requiring admission are of high severity. Typically, 45 minutes are spent at the bedside and on the patient's facility floor or unit. |
| HCPCS | 99307 | Subsequent nursing facility care, per day, for the evaluation and management of a patient, which requires at least 2 of these 3 key components: a problem focused interval history; a problem focused examination; straightforward medical decision making. Counseling and/or coordination of care with other physicians, other qualified health care professionals, or agencies are provided consistent with the nature of the problem(s) and the patient's and/or family's needs. Usually, the patient is stable, recovering, or improving. Typically, 10 minutes are spent at the bedside and on the patient's facility floor or unit. |
| HCPCS | 99308 | Subsequent nursing facility care, per day, for the evaluation and management of a patient, which requires at least 2 of these 3 key components: an expanded problem focused interval history; an expanded problem focused examination; medical decision making of low complexity. Counseling and/or coordination of care with other physicians, other qualified health care professionals, or agencies are provided consistent with the nature of the problem(s) and the patient's and/or family's needs. Usually, the patient is responding inadequately to therapy or has developed a minor complication. Typically, 15 minutes are spent at the bedside and on the patient's facility floor or unit. |
| HCPCS | 99309 | Subsequent nursing facility care, per day, for the evaluation and management of a patient, which requires at least 2 of these 3 key components: a detailed interval history; a detailed examination; medical decision making of moderate complexity. Counseling and/or coordination of care with other physicians, other qualified health care professionals, or agencies are provided consistent with the nature of the problem(s) and the patient's and/or family's needs. Usually, the patient has developed a significant complication or a significant new problem. Typically, 25 minutes are spent at the bedside and on the patient's facility floor or unit. |
| HCPCS | 99310 | Subsequent nursing facility care, per day, for the evaluation and management of a patient, which requires at least 2 of these 3 key components: a comprehensive interval history; a comprehensive examination; medical decision making of high complexity. Counseling and/or coordination of care with other physicians, other qualified health care professionals, or agencies are provided consistent with the nature of the problem(s) and the patient's and/or family's needs. The patient may be unstable or may have developed a significant new problem requiring immediate physician attention. Typically, 35 minutes are spent at the bedside and on the patient's facility floor or unit. |
| HCPCS | 99315 | Nursing facility discharge day management; 30 minutes or less |
| HCPCS | 99316 | Nursing facility discharge day management; more than 30 minutes |
| HCPCS | 99318 | Evaluation and management of a patient involving an annual nursing facility assessment, which requires these 3 key components: a detailed interval history; a comprehensive examination; and medical decision making that is of low to moderate complexity. Counseling and/or coordination of care with other physicians, other qualified health care professionals, or agencies are provided consistent with the nature of the problem(s) and the patient's and/or family's needs. Usually, the patient is stable, recovering, or improving. Typically, 30 minutes are spent at the bedside and on the patient's facility floor or unit. |
| VHA Stop Code | 119 | Visit by VA professional/clinical staff [usually Registered Nurse (RN), Social Worker (SW), Case Manager (CM)] for assessment of a Veteran receiving Long Term Services and Supports (LTSS) in a community nursing home (CNH). The purpose of the visit is to evaluate the resident's health status, treatment plan, and continued need for CNH services. |
| VHA Stop Code | 121 | Records visit by VA professional/clinical staff (usually RN, SW, CM) for assessment of a Veteran receiving Long Term Services and Supports (LTSS) residing in a VA approved Community Residential Care (CRC) home (includes Medical Foster Homes (MFH), Assisted Living, Personal Care Homes, Family Care Homes, and psychiatric CRC Homes). VA CRC staff makes referrals to the facilities in which the Veterans reside but the Veteran pays for her/his own care. |
| VHA Stop Code | 651 | Records number of State Nursing Home (SNH) days which the patient had in a VA-paid, State-provided SNH for that month. |

**Supplemental Table 8:**  Remote Monitoring-Related Abnormalities Expected to Lead to Possible Clinical Action

| **Remote transmission demonstrating notifiable arrhythmia** |
| --- |
| AFL/AF episodes noted |
| AFL/AF noted, longest episode 30 sec to < 6 minutes |
| AFL/AF noted, longest episode 6 minutes to < 24 hours |
| AFL/AF noted, longest episode >=24 hours |
| AFL/AF noted, persistent (> 7 days) |
| Treated AT/AF episode(s) |
| VT/VF on the presenting electrogram |
| VT detected in the monitor zone |
| Inappropriate VT/VF detection in a monitor zone |
| VT/VF inappropriately called SVT by discriminators |
| Appropriate discrimination of SVT |
| High V rate episodes |
| High V rate episodes, non-sustained VT |
| High V rate episodes, SVT |
| High V rate episodes, AFL/AF with rapid ventricular response |
| High V rate episodes, oversensing |
| Agonal rhythm |
| BiV pacing <90% |
| Battery at ERI |
| Battery at EOL |
| RV lead impedance out of range |
| LV lead impedance out of range |
| Atrial lead impedance out of range |
| HV/SVC impedance out of range |
| Possible RV failure to capture |
| Possible LV failure to capture |
| Possible atrial lead failure to capture |
| Possible lead failure |
| Ventricular undersensing |
| Atrial undersensing |
| Ventricular oversensing |
| Atrial oversensing |
| Possible generator failure |
| VT/VF with ATP |
| VT/VF with shock(s) |
| VT/VF with ATP and shock(s) |
| Inappropriate ATP therapy |
| Inappropriate shock therapy (+/- ATP) |

Abbreviations: atrial fibrillation (AF), atrial flutter (AFL), antitachycardia pacing (ATP), biventricular (BiV), end of life (EOL), elective replacement indicator (ERI), left ventricular (LV), right ventricular (RV), supraventricular tachycardia (SVT), ventricular (V), ventricular fibrillation (VF), ventricular tachycardia (VT)
